# Functional Capacity of Shiga-Toxin Promoter Sequences in Eukaryotic Cells

**DOI:** 10.1371/journal.pone.0057128

**Published:** 2013-02-22

**Authors:** Leticia V. Bentancor, Marcos F. Bilen, María P. Mejías, Romina J. Fernández-Brando, Cecilia A. Panek, Maria V. Ramos, Gabriela C. Fernández, Martín Isturiz, Pablo D. Ghiringhelli, Marina S. Palermo

**Affiliations:** 1 División Inmunología, Instituto de Medicina Experimental (IMEX) (CONICET), Academia Nacional de Medicina, Buenos Aires, Argentina; 2 Laboratorio de Ingeniería Genética y Biología Celular y Molecular, Universidad Nacional de Quilmes, Buenos Aires, Argentina; University of Helsinki, Finland

## Abstract

Shiga toxins (Stx) are the main virulence factors in enterohemorrhagic *Escherichia coli* (EHEC) infections, causing diarrhea and hemolytic uremic syndrome (HUS). The genes encoding for Shiga toxin-2 (Stx2) are located in a bacteriophage. The toxin is formed by a single A subunit and five B subunits, each of which has its own promoter sequence. We have previously reported the expression of the B subunit within the eukaryotic environment, probably driven by their own promoter. The aim of this work was to evaluate the ability of the eukaryotic machinery to recognize *stx2* sequences as eukaryotic-like promoters. Vero cells were transfected with a plasmid encoding Stx2 under its own promoter. The cytotoxic effect on these cells was similar to that observed upon incubation with purified Stx2. In addition, we showed that Stx2 expression in Stx2-insensitive BHK eukaryotic cells induced drastic morphological and cytoskeletal changes. In order to directly evaluate the capacity of the wild promoter sequences of the A and B subunits to drive protein expression in mammalian cells, GFP was cloned under eukaryotic-like putative promoter sequences. GFP expression was observed in 293T cells transfected with these constructions. These results show a novel and alternative way to synthesize Stx2 that could contribute to the global understanding of EHEC infections with immediate impact on the development of treatments or vaccines against HUS.

## Introduction

Shiga toxins (Stx) are the main virulence factors in enterohemorrhagic *Escherichia coli* (EHEC) infections, causing diarrhea, hemorrhagic colitis, and hemolytic uremic syndrome (HUS). The infection is associated with the ingestion of contaminated meat or vegetables but is also transmitted by water or even person-to-person contact [1]–[3]. Sporadic or massive outbreaks have been reported in several developing countries. In Argentina, HUS is endemic and represents a serious public health problem with high morbidity and mortality rates [4], [5].

Shiga toxin is a member of the AB5 family of bacterial toxins. The A subunit (StxA) possesses N-glycosidase activity against 28S rRNA of 60S ribosomes in the cytosol, resulting in inhibition of protein synthesis in eukaryotic cells. The five B subunits (StxB) form a pentamer that binds to globotriaosyl ceramide receptors (Gb_3_) on the cell membrane [6]. Stx-producing *E. coli* (STEC) express two types of Stx proteins (Stx1 and Stx2) and their variants, being Stx2 more virulent and epidemiologically more relevant than Stx1.

In most of the STEC strains identified, the toxin genes, *stxAB,* are located in the genomes of prophages that resemble the coliphage lambda [7]. The lytic phase, which is induced under stress conditions, leads to an enhancement of Stx2 production and release [8]–[10]. In this stage, the viral progeny is able to infect other bacteria present in the gut [11], [12]. It has been demonstrated that Stx phages can survive even after host death. Moreover, under convenient circumstances, the phage may transduce *in vivo* and *in vitro* other bacteria [13]. In fact, Shiga toxin-converting bacteriophages are able to infect and lysogenize laboratory strains of *E. coli* as well as *E. coli* strains derived from the human intestine [14]. The resulting lysogenic strains are able to produce toxins and infectious phage particles, facilitating the spread of toxin genes among *E. coli* strains and other *Enterobacteriaceae* [14].

On the other hand, different lines of evidence have shown that bacteriophage lambda is able to transduce mammalian cells, and that bacteriophage lambda vectors containing a mammalian gene expression cassette are able to express encoded genes in mammalian target cells *in vitro* [15]–[17] and *in vivo* [18], [19]. The ability of lambda phage particles to transduce mammalian cells *in vivo* depends on the phagocytic and nonphagocytic uptake of the phage, possibly including macropinocytosis, and is increased through an Fc receptor-mediated antibody-dependent mechanism [20].

The interaction between EHEC and macrophages has been reported and it has been shown that phagocytosis of EHEC by murine macrophages causes actin rearrangements surrounding the phagosome. Intracellularly produced Stx has been shown to be responsible for these effects [21]. In the same line of evidence, a correlation between the *E. coli* O157:H7 phagocytosis by THP-1 human macrophages and the presence of Stx within the cells has been recently described. In addition, *stxA* and *stxB* transcription in infected macrophages and upregulation of SOS response genes (such as *recA*, *recC*, and *recN*) occur simultaneously [22]. We analyzed the hypothesis that, independently of the mechanism through which bacterial *stx* genes are delivered into mammalian cells during EHEC intestinal infection, eukaryotic cells are able to transcribe a functionally active Stx-like protein. In a previous report, BHK cells transfected with a DNA vaccine carrying the wild-type promoters of Stx2 were able to express both subunits. B subunit expression probably reflected the presence of eukaryotic putative promoter-like sequences located upstream of it [23].

Therefore, the aim of this study was to evaluate the ability of the eukaryotic machinery to recognize genetic sequences as *stx2* promoters, to transcribe a Stx2-like protein and produce the functionally active toxin. For this purpose, we designed plasmid constructions using green fluorescent protein (GFP) under putative promoter-like sequences located upstream of the open reading frames (ORFs) of *stxA* and *stxB*, and tested them for GFP expression. In addition, we transfected two eukaryotic cell lines with different sensitivity profiles to Stx2, Vero and BHK cells, with a plasmid construction carrying the *stx2* gene under its own promoter. In both cells we confirmed the Stx2-specific cytotoxic effect.

These results suggest the existence of a new pathway in Stx2 production. In the context of the inflammatory response that takes place in the gut, the phagocytic cells (macrophages and neutrophils) could uptake *stx* genes, produce the active toxin and release it into the bloodstream. Although immune cells may produce low doses of Stx2, they may play an important role in the EHEC pathogenesis and in the global course of infection. In order to develop an efficient procedure for the treatment of EHEC infections, it is necessary to understand the mechanisms involved in the expression of the genes encoding Shiga toxins. In this regard, the present study may open new avenues for the treatment and prevention of HUS.

## Results

### *In silico* Analysis

**Figure 1:**
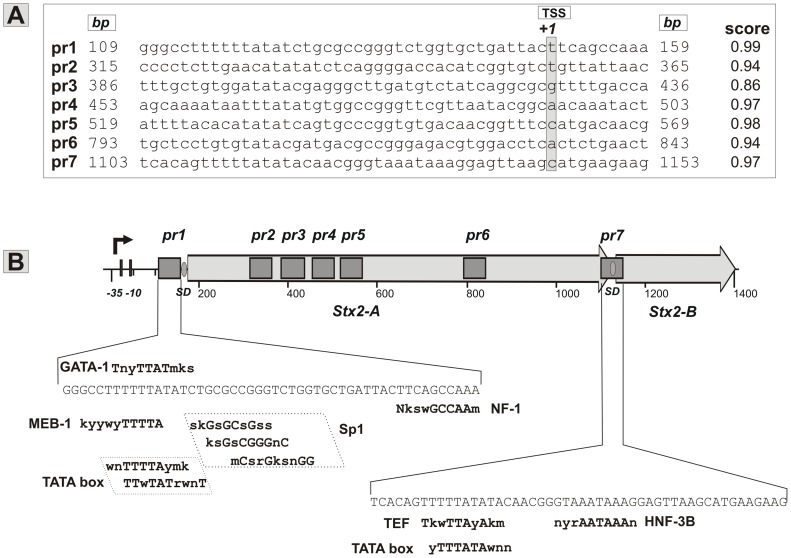
*In silico* analysis of the *stx*2 sequence. **A.** Seven regions (pr1-7) with high score for putative eukaryotic promoter sequences were found. The putative transcription start site (TSS, +1) detected with promoter prediction server is highlighted in gray **B.** The pr1-pr7 regions are indicated with dark gray boxes over the *stx2* gene. Putative mammalian transcription factor binding sites are indicated on the corresponding sequences of the pr1 and pr7 regions.

We analyzed the whole sequence of *stx2* including the promoter region by means of several softwares to find putative eukaryotic promoter sequences. Seven putative eukaryotic promoter-like sequences were computationally detected (Figure 1). Based on their position, pr1 and pr7 putative promoter sequences were additionally analyzed to find transcription factor binding sites. The pr1 sequence contains five eukaryotic transcription factor binding sites, MEB-1, GATA-1, TATA box, Sp1 and NF-1, whereas the pr7 sequence contains three eukaryotic transcription factors binding sites, TEF, TATA box and HNF-3B. The protein factors that bind to these sequences could act together with RNA polymerase to initiate a transcriptional process [24].

### Pr1 and Pr7 Sequences are Recognized by Eukaryotic Cells

**Figure 2:**
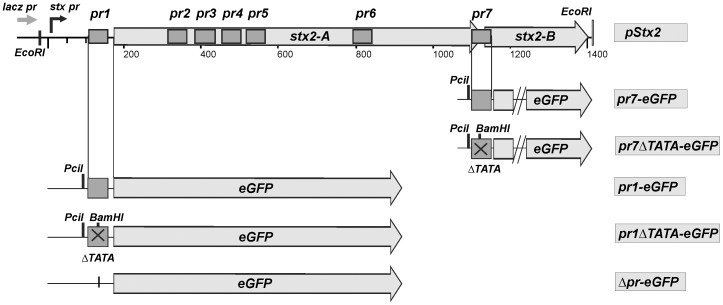
Recombinant plasmid constructs. pStx2 is the pGEM-T plasmid with the *stx2* locus cloned in the same direction that *lacZ* promoter. pr1-eGFP and pr7-eGFP are reporter plasmids with the region pr1 or pr7 driving eGFP expression, and Δpr-eGFP is a construction lacking the wild CMV promoter and was used as negative control. Linear pr1-eGFP and pr7-eGFP were obtained after digestion with PciI. In pr1ΔTATA-eGFP and pr7ΔTATA-eGFP TATA Box sequence has been replaced by BamHI restriction site. *Stx2* linear DNA was obtained after digestion with EcoRI.

The pr1 and pr7 sequences were selected to further analyze the promoter activity in the eukaryotic environment. The pr1-eGFP and pr7-eGFP reporter plasmids were constructed to test whether the prokaryotic sequences could drive the transcription of the *gfp* gene (Figure 2). These plasmids were used in circular and linearized forms and pEGFP-N3 and Δpr-eGFP were used as positive and negative controls, respectively.

**Figure 3:**
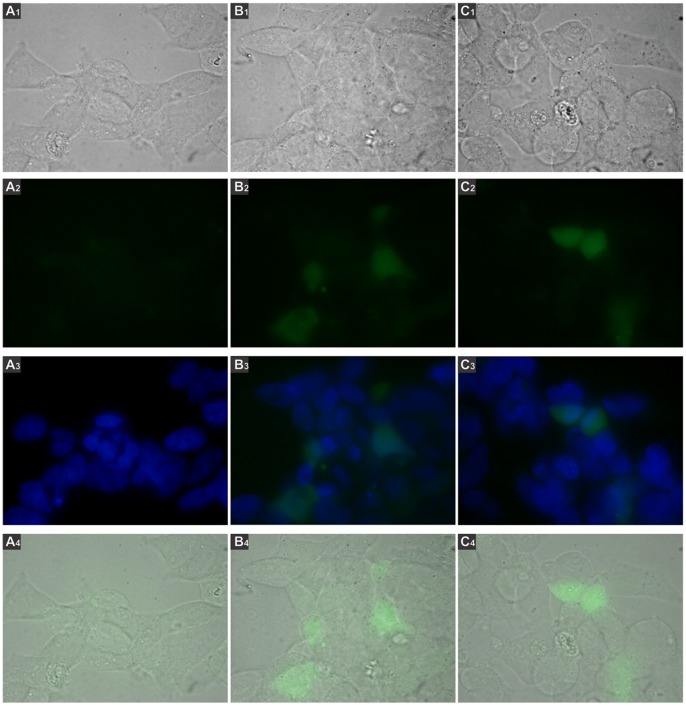
GFP activity driven by the pr1 or pr7 regions. 293 T cells were transfected with plasmids pr1-eGFP, pr7-eGFP or Δpr-eGFP, incubated for 48 h and analyzed by fluorescence microscopy using the Nikon Eclipse TE2000 microscope equipped with a CCD camera, with 1000X magnification. Green fluorescence photos were taken with 400 ms of exposure and 3.2 of gain. Numbers 1, 2, 3, 4 correspond to images visualized with white light, green filter, merge between DAPI and green filter and merge between white light and green filter, respectively. **A.** Cells transfected with the Δpr-eGFP plasmid. **B.** Cells transfected with pr1-eGFP. **C.** Cells transfected with pr7-eGFP.

**Figure 4:**
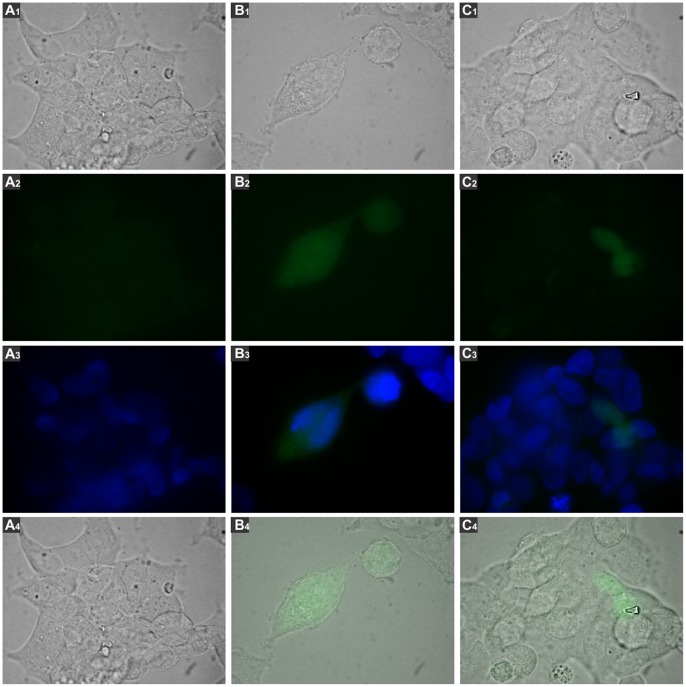
GFP activity driven by linear reporter plasmid. 293 T cells were transfected with pr1-eGFP or pr7-eGFP linearized with Pcil restriction enzyme. After 48 h, cells were analyzed by fluorescence microscopy using Nikon Eclipse TE2000 microscope equipped with a CCD camera, using 1000X magnification. Green fluorescence photos were taken with 400 ms of exposure and 3.2 of gain. Numbers 1, 2, 3, 4 correspond to images visualized with white light, green filter, merge between DAPI and green filter and merge between white light and green filter, respectively. **A.** 293 T cells transfected with the Δpr-eGFP plasmid. **B.** Cells transfected with linear pr1-eGFP. **C.** Cells transfected with linear pr7-eGFP.

293T cells were transfected with the constructions and GFP expression was analyzed by fluorescence microscopy. We observed GFP expression when cells were transfected with pr1-eGFP (Figure 3, panel B) or pr7-eGFP (Figure 3, panel C). In contrast, no fluorescence was detected when 293T cells were transfected with the Δpr-eGFP plasmid (Figure 3, panel A). In addition, GFP expression was observed when cells were transfected with the linearized forms of pr1-eGFP or pr7-eGFP (Figure 4 panels B and C respectively). These results demonstrate that pr1 and pr7 are the sequences used by mammalian to drive GFP expression.

To evaluate the relevance of TATA box present at pr1 and pr7 sequence, a mutational assay was done. The six nucleotides of the TATA box core were replaced by a BamHI restriction site giving pr1ΔTATA-eGFP or pr7ΔTATA-eGFP [Figure 2]. GFP expression was not observed when 293T cells were transfected with pr1ΔTATA-eGFP or pr7ΔTATA-eGFP (Figure S1, panels A and B respectively), suggesting that this sequence plays a critical role in the promoter activity of the putative promoters.

### Eukaryotic Cells Transfected with pStx2 are Able to Express Stx2 Protein

**Figure 5:**
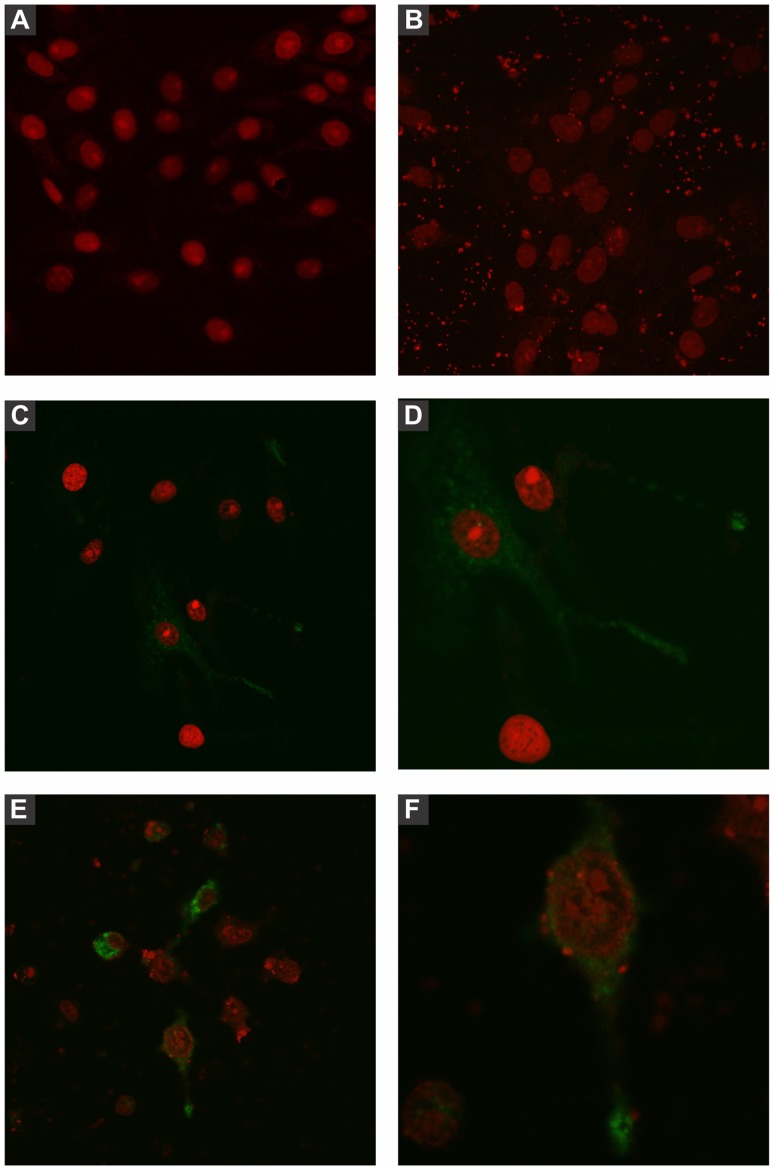
Immunofluorescence assay of Vero cells incubated with Stx2. Cells were incubated with purified Stx2 (100 CD50) or transfected with the pStx2 plasmid and stained with propidium iodide. Presence of Stx2 was detected with polyclonal anti-Stx2 serum and FITC-conjugated goat anti-mouse antibody, and analyzed by confocal microscopy. Original magnification 600X. **A:** Non-treated Vero cells. **B:** Cells transfected with the pGEM-T vector alone. **C:** Cells incubated with purified Stx2. **D:** Digital zoom (3.5X) of picture C. **E:** Cells transfected with pStx2. **F:** Digital zoom (3.5X) of picture **E**.

To evaluate the expression of the Stx2 protein in the eukaryotic environment, Vero cells were transfected with pStx2, and intracytoplasmic Stx2 was revealed after 48 hours by an indirect immunofluorescence assay using a polyclonal anti-Stx2 antibody (Figure 5). Cells incubated with Stx2 (Figure 5, C/D) or cells transfected with pStx2 (Figure 5, E/F) shown similar specific staining. No fluorescence was detected in Vero cells without treatment (Figure 5A) or transfected with the empty plasmid (Figure 5B).

### Stx2 Expressed by Eukaryotic Cells is Biologically Active

We then evaluated the Stx2-specific cytotoxicity after pStx2-transfection of Vero cells, as a representative Stx2-susceptible cell line. Cells incubated with purified Stx2 and cells transfected with the empty plasmid were used as positive and negative controls, respectively. Non treated cells were also analyzed.

**Figure 6:**
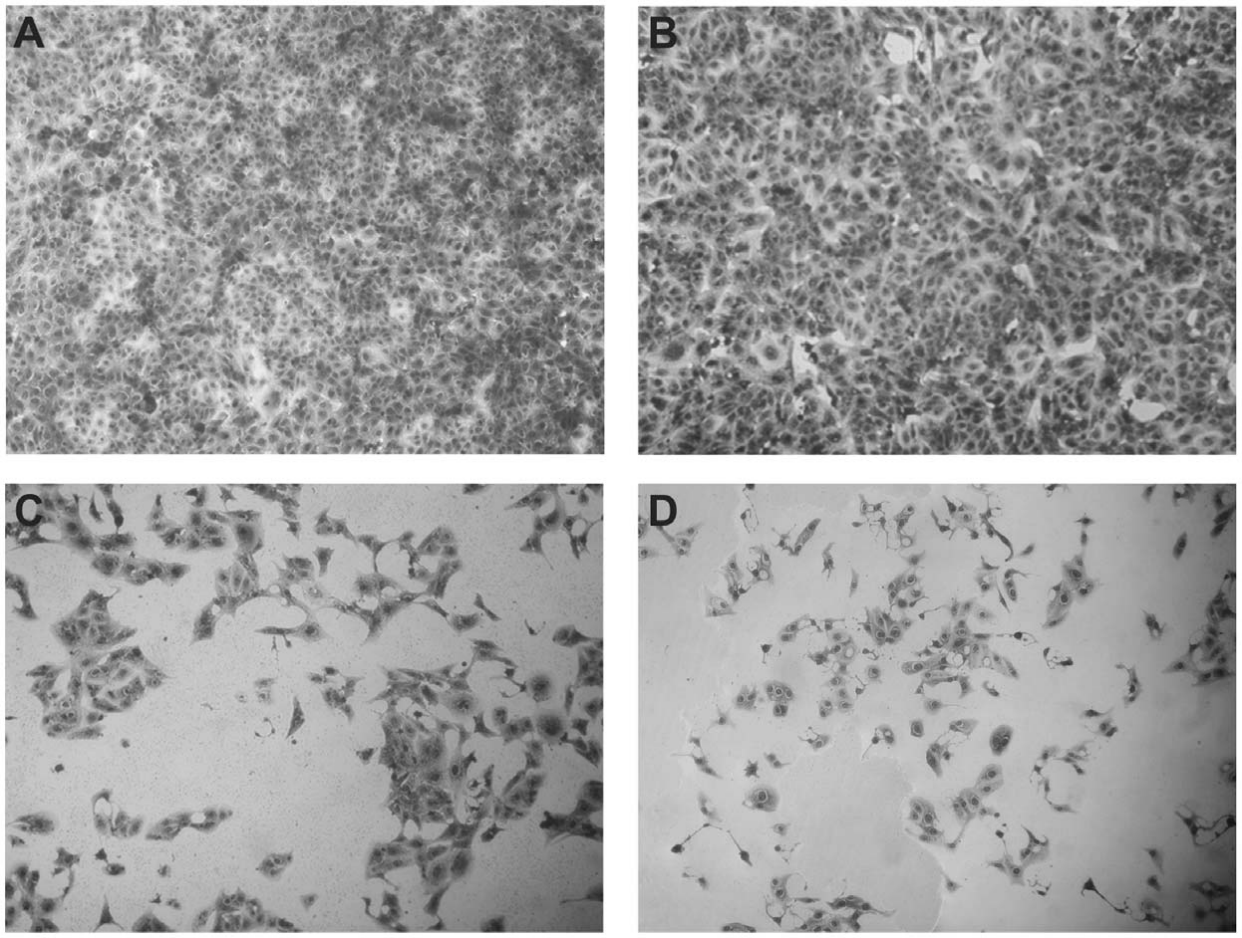
Cytotoxicity on Vero Cells. Vero cells were incubated with purified Stx2 (1 CD50) or transfected with pStx2 plasmid. After 48 h, cells were stained with Crystal Violet and analyzed by optical microscopy. Representative pictures using 200X original magnification are shown. **A.** Non-treated Vero cells. **B.** Cells transfected with the pGEM-T plasmid. **C.** Cells transfected with the pStx2 plasmid. **D.** Cells incubated with purified Stx2.

After 48 hours, Vero cells transfected with pStx2 (Figure 6C) showed a similar cytotoxicity to that of cells incubated with 1 CD50 of purified Stx2 (Figure 6D). No cytotoxic effects were observed in non-treated Vero cells (Figure 6A) or transfected with control plasmid (Figure 6B).

**Figure 7:**
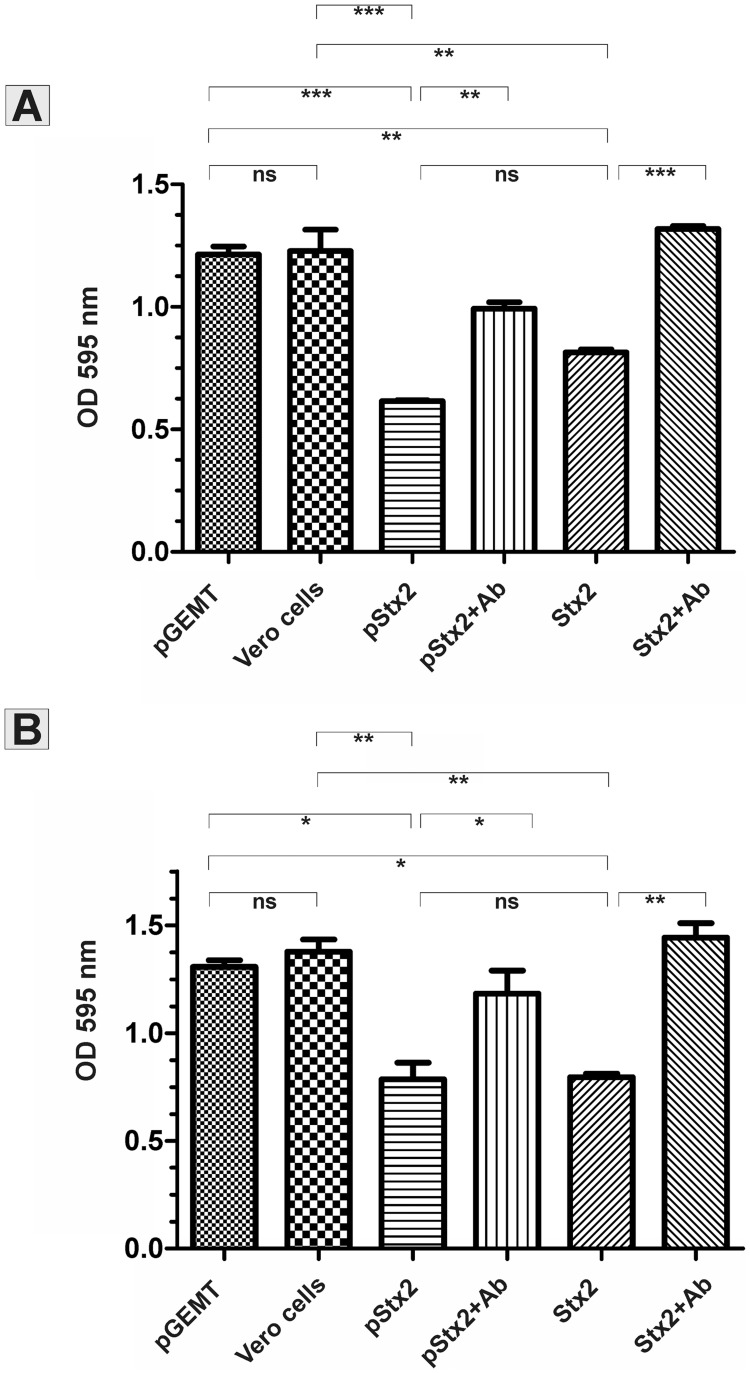
Neutralization of the Stx2 cytotoxic activity. Vero cells were incubated with a 1∶1600 dilution of cellular extracts (Panel A) or a 1∶400 dilution of culture supernatants (Panel B) derived from Vero cells transfected with pGEM-T (pGEM-T) or pStx2 (pStx2). As positive and negative controls, Vero cells were incubated with 1 CD50 of Stx2 (Stx2) or in medium (Vero cells), respectively. To evaluate the specificity of the cytotoxicity, cytotoxic samples were pre-incubated with mouse polyclonal anti-Stx2 antibodies (pStx2+Ab; Stx2+Ab). After 48 h, cells were stained with Crystal Violet and OD_595_ was measured as detailed in Materials and Methods. One-way ANOVA (Tukey’s Multiple Comparison Test) was used to determine statistical significance between different samples.*P<0.05. **P<0.01. ***P<0.001.

In order to further analyze the cytotoxicity induced by pStx2, non-treated (naïve) Vero cells were incubated for 48 hours with supernatants or cellular extracts derived from Vero cells transfected with pStx2, or with the empty plasmid as control. Both the supernatants and cellular extracts obtained from pStx2-transfected cells showed cytotoxic effect on naïve Vero cells (Figure 7). No cytotoxicity was observed in non-treated Vero cells or Vero cells incubated with supernatants or extracts from Vero cells transfected with the control plasmid. Significant neutralization of the cytotoxic activity was observed when supernatants or cellular extracts were incubated with mouse polyclonal anti-Stx2 antibodies, confirming that the cytotoxicity was specifically induced by Stx2 (Figure 7). To rule out cryptic promoter activity in the pGEMT backbone, pStx2 was digested with EcoRI and the fragment containing *stx2* coding sequence alone was gel purified and used to transfect Vero cells. After 48 hours, Vero cells transfected with the linear DNA still showed cytotoxicity. However, quantification of cytotoxicity revealed that it was two-fold less cytotoxic than its counterpart pStx2 (data not shown).

### Stx2 Expressed by BHK Cells Induces Morphological Changes

**Figure 8:**
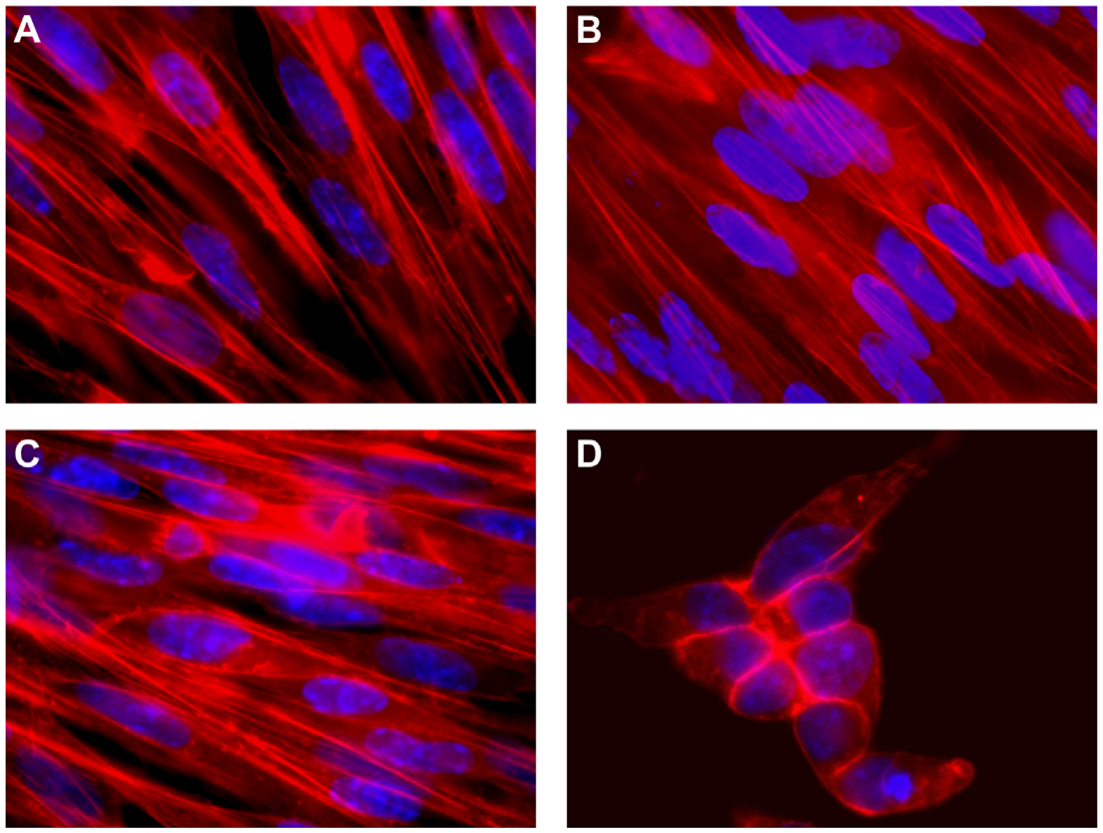
Cytotoxicity on BHK cells. BHK cells were incubated with purified Stx2 (1 CD50) or transfected with the pStx2 plasmid. After 48 h, cells were stained with DAPI and phalloidin-TRITC and analyzed by fluorescence microscopy. Original magnification 600X. **A.** Non-treated BHK cells. **B.** Cells transfected with pGEM-T. **C.** Cells incubated with purified Stx2. **D.** Cells transfected with the pStx2 plasmid.

BHK cells transfected with pStx2 showed morphological alterations and changes in their growth as evaluated by optical microscopy. BHK cells transfected with pStx2 increased their intracellular spaces and the cells became rounder and smaller. However, cells transfected with the control plasmid or non-treated cells did not shown morphological changes. To further analyze pStx2 effects on actin and nucleic acids on BHK cells, fluorescence assays were performed using phalloidin-TRITC and DAPI, respectively. Cytoskeleton changes were observed 48 hours after transfection. pStx2-transfected BHK cells showed filopodia-like and lamellipodia-like structures (Figure 8D), as Takenouchi and collaborators previously reported in ACHN cells treated with Stx1B [25].

We did not observe these effects in BHK cells without treatment (Figure 8A) or in those transfected with the control plasmid (Figure 8B). No effect was observed in BHK cells incubated with Stx2 (Figure 8C).

### Transduction of THP-1 Cells Differentiated to Macrophages by Bacteriophage 933W

**Figure 9:**
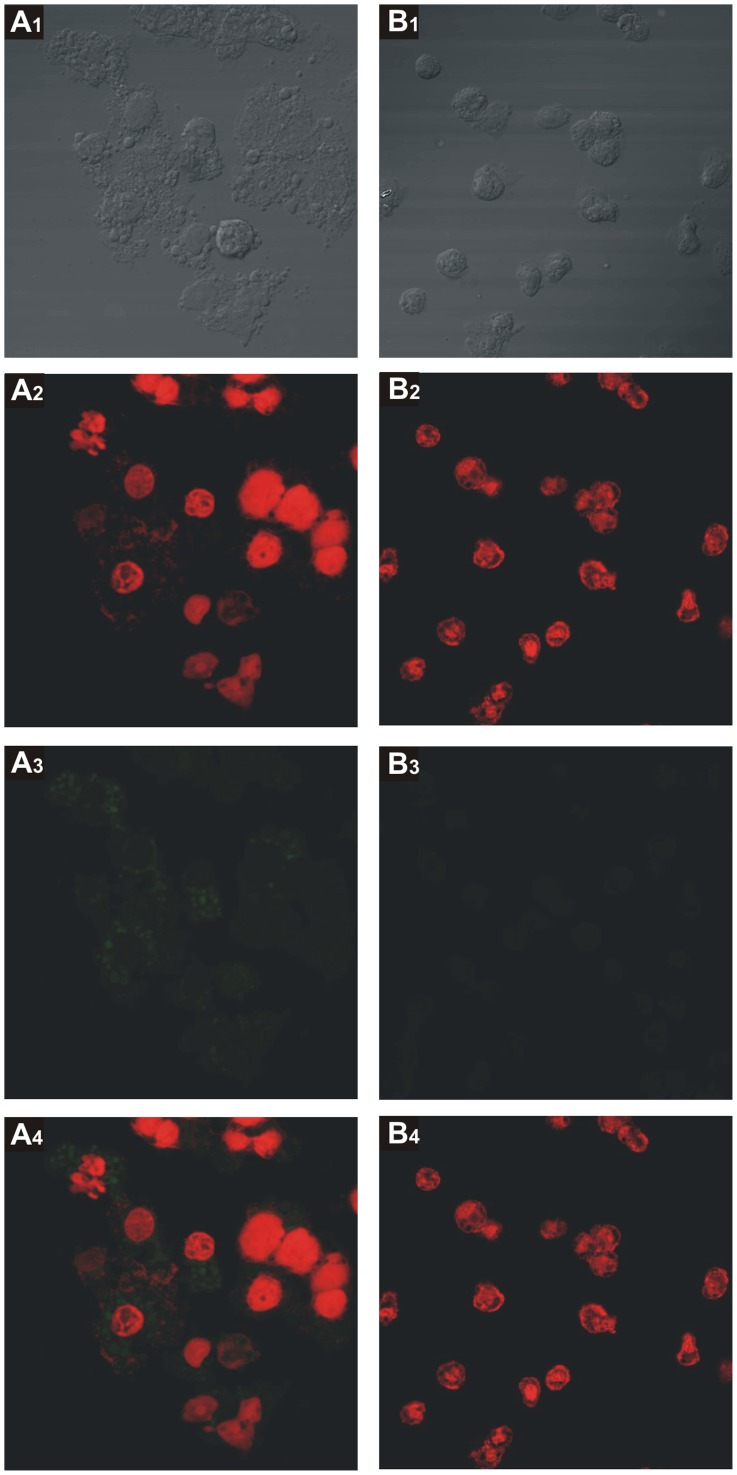
Transduction of THP-1 cells differentiated to macrophages by bacteriophage 933W. THP-1 cells PMA-differentiated to macrophages were transduced with φΔTOX-GFP. After 3 h, cells were analyzed by confocal microscopy, using 600X magnification. Green fluorescence photos were taken with 400 ms of exposure and 1 of gain. Numbers 1, 2, 3, 4 correspond to images visualized with white light, red filter, green filter and merge between green and red filter, respectively. **A.** Cells transduced with φΔTOX-GFP. **B.** Non-treated cells.

To analyze the hypothesis that 933W bacteriophage is able to transduce mammalian cells *in vivo,* THP-1 cells PMA-differentiated to macrophages were incubated with purified 933W bacteriophage in which *stx2* gene was replaced by *gfp* sequence (φΔTOX-GFP) [14]. We observed GFP expression when macrophages were incubated with φΔTOX-GFP (Figure 9, panel A). No fluorescence was observed in non-treated macrophages (Figure 9, panel B).

## Discussion

The main finding of the present article was the demonstration of the existence of putative eukaryotic promoter-like sequences located upstream of the genes encoding for the Stx2 A and B subunits. Moreover, the eukaryotic machinery was able to recognize these sequences and to initiate the transcription and translation leading to the synthesis of functional active Stx2-like protein.

Production of Shiga toxins is generally associated with prophage induction, which depends, among other factors, on the location of the integration site in the bacterial genome, which in turn depends on the phage type and bacterial host. In addition, it depends on the activation of the bacterial RecA protein, which causes autocleavage of the cI repressor encoded by the phage [26], [27]. This mechanism allows the transcription from phage promoters, in addition to the p_R_’ promoter, which controls the transcription of the *stxA* and *stxB* genes. In this context, factors that damage DNA or inhibit replication of the bacterial genome can activate the SOS system, and lead to prophage induction and production of large quantities of toxins.

Many of the prophages found in *E. coli* O157 strains are lambdoid phages, two of which, Sp15 and Sp5, carry the *stx1* and *stx2* genes, respectively [28]. In the prototype of the lambdoid family, lambda phage, Int and Xis proteins are needed to produce an efficient excision of genomic DNA from the bacterial genome both *in vivo* and *in vitro* [29].

It is known that prophage release/replication and toxin production are correlated. One of the physiological extracellular signals that induce prophage activation is the presence of hydrogen peroxide released from human neutrophils after lysogenic bacteria uptake [30], [28]. The excision process triggers the replication cascade and the generation of phage progeny. Furthermore, within the phagocytic vesicles, bacteria are under stress and the lytic cycle is activated. Some excised DNA molecules can be released into the cytoplasm from the phagocytic vesicle or phagolysosome, and then be incorporated to the nucleus. Otherwise, the free phage could transduce eukaryotic cells and the DNA phage could be transported to the nucleus [19].

On the other hand, several reports have demonstrated that bacteria could transfer plasmid-encoded genes to both bacterial and eukaryotic cells. In particular, gene transfer from *Salmonella* to mammalian macrophages and dendritic cells has been observed [31]–[33]. Likewise, when the lytic cycle of phages is activated in the phagolysosome after bacterial phagocytosis by macrophages, transfer of *stx* genes may occur [30], [34]. Thus, intracellularly released DNA phages could use either one or both mechanisms of gene delivery, via the viral particle or by the naked DNA phage. Both of these mechanisms might transfer the *stx* gene to the cellular nuclei where it can be transcribed. In either situation, the eukaryotic transcriptional machinery could recognize the DNA sequences encoding Stx2 toxins. Binding motifs for eukaryotic transcription factors detected in the sequence of *stx2* and described in this article support this hypothesis.

In the present work, we explored the ability of mammalian cells carrying *stx2* coding DNA to produce Stx2, constituting an alternative Stx2 source during the course of the EHEC infection.

In a previous report, we have shown that the B subunit of Stx2 is expressed in the eukaryotic environment without the need of an additional eukaryotic promoter, involving probably its own upstream sequences [23].

In mammalian cells, transcriptional regulation requires the cooperation of multiple factors and specific sequences (cis-regulatory elements), including promoters, enhancers and silencers. The structure of the eukaryotic core promoter contains the TATA box, BRE (TFIIB recognition element), MTE (Motif Ten Element), DPE (Downstream Promoter Element), Inr (Initiator) and DCE (Downstream Core element) sequences, among others. Very often, mammalian core promoters contain CG-rich sequences called CpG islands or a mix of different motifs. The Sp1 binding site is often found in this type of core promoters [35].

The initiation of transcription in the eukaryotic environment can occur over a single start site (focused core promoter) or over distinct start sites into a short region (dispersed core promoter), depending on which binding motifs are present. Focused core promoters are mainly related to the TATA box, Inr and DPE motifs, whereas dispersed core promoters are related to CpG island motifs [36]–[39].

*In silico* analyses of the *stx2* gene suggest the presence of different typical core promoter regulatory sequences. We focused only in the putative promoter sequences pr1 and pr7 that are located upstream of the *stx2A* and *stx2B* ORFs respectively (Figure 1), because if functional, they allow the synthesis of the complete sequences of A and B subunits respectively. A detailed bioinformatic analysis using transcription factor binding site databases and different servers led to the detection of several eukaryotic transcription factor binding motifs. In the pr1 putative promoter sequence, we detected five binding sites: MEB-1, GATA-1 and TATA box (partially overlapped with each other), and Sp1 and NF-1 (overlapped with TSS) (Figure 1). In the pr7 putative promoter sequence, we detected three binding sites: TEF and TATA box (partially overlapped), and HNF-3B. Based on this analysis, the pr1 and pr7 sequences could be acting as putative sequences in a eukaryotic context with potential promoter activity for the A and B subunits, respectively. Monolayers of 293T cells transfected with pr1-eGFP and pr7-eGFP showed GFP expression, demonstrating that eukaryotic cells should recognize both sequences as functional promoters. Although TATA box is not the only one eukaryotic transcription factor binding sites found, it is known that TBP (TATA binding protein) along with other TBP-associated factors, make up the transcription Factor II D (TFIID), a general transcription factor that in turn makes up part of the RNA polymerase II pre-initiation complex [40]. Because TBP is one of the few proteins in the pre-initiation complex that binds DNA in a sequence-specific manner, it helps to position RNA polymerase II over the transcription start site of the gene. GFP expression was not observed when we transfected 293T cells with pr1ΔTATA-eGFP or pr7ΔTATA-eGFP, suggesting that this sequence plays a critical role in the promoter activity of the putative promoters. However, these results are not enough to attribute to TATA box the total role in driving Stx2 expression. Further studies will be necessary to fully define the role of the other mammalian transcription factor binding sites present in the Stx2 sequence.

Moreover, Vero cells transfected with pStx2 produced an active Stx2-like protein that was specifically detected or neutralized by anti-Stx2 antibodies. In addition, the linear *stx2* sequence was still able to induce cytotoxicity in Vero cells, discarding the presence of a cryptic promoter activity in the pGEMT backbone. The less cytotoxicity observed comparing the linear s*tx2* sequence with pStx2 is probably due to the lower efficiency of transfection between linear DNA sequences and supercoiled plasmid.

It is known that Stx2 binds to its specific receptor on mammalian cells, the globotriaosyl ceramide (Gb_3_), to exert its cytotoxic effects. However, Gb_3_ expression is insufficient to confer sensitivity to Stx2, implying that other key factors, such as toxin-receptor internalization or intracellular degradation, are important for its effect [41]. The data published so far suggest that Stx2 toxins need to traverse the cell membrane to exert their effects on protein synthesis.

The toxin binds to Gb_3_ receptors and it is internalized through clathrin-dependent and -independent mechanisms. Once internalized, the toxin can follow one of several routes. The fatty acid chain composition in the receptor, rather than the toxin itself, contains the trafficking signal necessary to translocate the toxin from the membrane to the cytosol. Along this intracellular way, the A and B subunits of Stx2 disassociate, unfold, and the cleavage of the A subunit into the A1 and A2 subunits triggers its enzymatic activity [42], [43].

Vero and BHK cells transfected with pStx2 showed different cytotoxic profiles probably due to their different toxin sensitivity. Vero cells transfected with pStx2 showed high cytotoxicity, similar to that observed when cells were incubated with exogenous recombinant Stx2.

Moreover, supernatants and cytoplasmic fractions from Vero cells transfected with pStx2 were able to induce a specific cytotoxic effect when they were added to naïve Vero cells. These results suggest that Stx2 is synthesized and released by Vero cells, acting in autocrine and paracrine ways in other non-transfected Vero cells, probably through the interaction with the Gb_3_ receptor present in the membrane. Indeed, it is possible that different and alternative mechanisms were acting simultaneously.

On the other hand, BHK cells transfected with pStx2 showed no significant lysis, but a clear Stx2 cytotoxic effect was observed on the actin cytoskeleton. These results probably reflected the low sensitivity of BHK cells to exogenous Stx2, due to the low Gb_3_ expression and/or their different fatty acid composition, but showed the Stx2 effect when it is produced intracellularly. Similarly, Nakagawa *et al*. [44] also reported cytotoxic effects in NIH3T3 and HeLa cells transfected with the Stx1A or Stx1B subunits, both cloned into pcDNA3.1 vectors. However, they observed no morphological changes or DNA fragmentation upon the addition of recombinant Stx1B protein (monomeric form) to NIH3T3 and HeLa cell cultures. The cells transfected with the *stx1B* gene became apoptotic with DNA fragmentation, whereas the cells transfected with the s*tx1A* gene were found to be necrotic but without signs of DNA fragmentation.

Moreover, actin morphological changes in macrophages infected with Stx-producing *Escherichia coli* O157:H7 have also been described [45]. Interestingly, in this report the authors suggested that the Stx released by the bacteria directly into the cytoplasm is responsible for the actin rearrangement in macrophages.

In conclusion, the capacity of mammalian cells to transcribe and translate *stx2* genes could have important pathological implications. First, it could contribute to the entry of Shiga toxins into the bloodstream. Secondly, the large numbers of inflammatory cells (macrophages and/or neutrophils) present during the infection process may release biologically active Stx2 close to target endothelial cells. Additionally, as preliminar data, we observed that purified φΔTOX-GFP was able to transduce the differentiated macrophagic THP-1 human cell line supporting our hypothesis that 933W bacteriophage could be able to transduce mammalian cells *in vivo*.

Thus, this alternative mechanism would not require a high blood concentration of Stx2 to be effective. In this regard, it has been recently demonstrated that a concentration as low as 10 fM of Stx2 is able to induce ribosome damage and to modulate selected cell signaling pathways that change cellular functions [46]. Understanding host-pathogen interactions is fundamental to develop vaccines and new specific therapeutic agents.

## Materials and Methods

### *In silico* Analysis of the *stx*2 Sequence

In order to detect eukaryotic transcription factor binding sites in the *stx*2 sequence, the putative promoter was analyzed with Neural Network Promoter Prediction v2.2 (http://www.fruitfly.org/seq_tools/promoter.html), which contains standardized data sets of human and *Drosophila melanogaster* genes. The putative promoters pr1 and pr7 were analyzed with the following server: AliBaba2.1 (http://www.gene-regulation.com/pub/programs/Alibaba2). The analysis was performed using the binding sites collected in TRANSFAC 4.0 database and default parameters (Pairsim to known sites: 50; Mat. width in bp: 10; minimum number of sites: 4; Minimum matrix conservation: 75%; Similarity sequence to matrix: 1%; Factor class level: 4).

### Plasmid Constructions

#### pStx2

The *stx2* complete sequence was amplified by PCR from total DNA from *E. coli* O157:H7 C600 (933W), using the primers Stx2Fw (5′-GAATTCATTATGCGTTGTTAG-3′) and Stx2R (5′-GAATTCTCAGTCATTATTAAACTG-3′), both containing an EcoRI restriction site [23]. The resulting fragment (1413 bp) was cloned in a pGEMT easy vector (PROMEGA Inc), generating the plasmid pStx2. This plasmid was replicated in *E. coli* DH5α competent cells.

As an additional control pStx2 was digested using EcoRI and the *stx2* complete sequence was gel purified, and used to transfect Vero cells.

#### pr1-eGFP

The pr1 motif localized immediately upstream to the *stx2A* open read frame (ORF) was amplified by PCR from the pStx2 construction, using the primers Stx2Fw1 (5′-GCTCTAGACATGTCATATTTATTTACCAGGCTCGC-3′) and Stx2R1 (5′-CCCCAAGCTTATACAGGTGTTCCTTTTGGC-3′), containing restriction sites PciI-XbaI and HindIII respectively. PCR fragment was digested with PciI and HindIII. The plasmid pEGFP-N3 was digested with the same enzyme in order to eliminate the wild CMV promoter (Clontech Laboratories, CA, USA). PCR fragment and the plasmid, digested, were ligated and transformed in *E. coli* DH5α competent cells. The construction was digested with PciI restriction enzyme to obtain the linear sequence. All restriction enzymes used were purchased from Fermentas International Inc.

#### pr7-eGFP

The pr7 motif localized immediately upstream to the *stx2B* ORF was amplified by PCR from the pStx2 construction using the primers Stx2Fw7 (5′-GCTCTAGACATGTTACAGCTGCAGCGTTTCTGAAC-3′) and Stx2R7 (5′-CCCCAAGCTTTCTTCTTCATGCTTAACTCCT-3′), containing restriction sites PciI-XbaI and HindIII respectively. PCR fragment was digested with PciI and HindIII. The plasmid pEGFP-N3 was digested with the same enzyme in order to eliminate the wild CMV promoter (Clontech Laboratories, CA, USA). PCR fragment and the plasmid, digested, were ligated and transformed in *E. coli* DH5α competent cells. The construction was digested with the PciI restriction enzyme to obtain the linear sequence.

#### Δpr-eGFP

The plasmid pEGFP-N3 was digested with PciI and HindIII, filled in with the Klenow fragment (PROMEGA Inc.) and re-ligated as negative control.

#### pr1ΔTATA-eGFP and pr7ΔTATA-eGFP

Complementary oligonucleotides (Macrogen Inc., Seoul, Republic of Korea) in which TATA box sequence was replaced by BamHI restriction site were hybridized (R1-TATAf 5′-CATGTTACCAGGCTCGCTTTTGCGGGCCTTGGATCCATCTGCGCCGGGTCTGGTGCTGATTACTTCAGCCAAAAGGAACACCTGTATA-3′, R1-TATAr 5′- AGCTTATACAGGTGTTCCTTTTGGCTGAAGTAATCAGCACCAGACCCGGCGCAGATGGATCCAAGGCCCGCAAAAGCGAGCCTGGTAA-3′; R7-TATAf 5′- CATGTTACAGCTGCAGCGTTTCTGAACAGAAAGTCACAGTGGATCCATACAACGGGTAAATAAAGGAGTTAAGCATGAAGAAGAA-3′, R7-TATAr 5′- AGCTTTCTTCTTCATGCTTAACTCCTTTATTTACCCGTTGTATGGATCCACTGTGACTTTCTGTTCAGAAACGCTGCAGCTGTAA-3′). pr1-eGFP and pr7-eGFP were digested with PciI and HindIII and ligated to the pr1ΔTATA or pr7ΔTATA fragment obtaining pr1ΔTATA-eGFP and pr7ΔTATA-eGFP respectively.

### *Stx2* Preparation

The toxin was purified as reported previously [23]. Briefly, *E. coli* JM109 transformed with plasmid pGEMT-Stx2 was used to express Stx2 protein. Bacteria were grown in Luria broth overnight in the presence of ampicillin (50 µg/ml). Cells were broken by ultrasonic treatment, centrifuged and the supernatant was treated with ammonium sulfate at 70% saturation. The precipitate was collected by centrifugation, resuspended in 3 ml of phosphate-buffered saline (PBS), and dialyzed with the same buffer for 24 h. Total protein concentration was determined by standard methods.

Stx2 concentration was determined with Ridascreen® Verotoxin kit (R-biopharm AG, Darmstadt, Germany). We calculated that 1CD50 (cytotoxic dose that kills 50% of Vero cells) of Stx2 is equivalent to 670 pg of Stx2.

### Anti-Stx2 Polyclonal Antibodies

Balb/c mice were immunized with Stx2 toxoid. Stx2 toxoid was prepared by formalin treatment of Stx2 [47]. Briefly, 100 µg of Stx2 (Phoenix Lab, Tufts University, Boston, MA) was incubated overnight in 2% formalin and then dialyzed extensively against PBS. Mice were immunized with 5 µg of Stx2 toxoid emulsified in Freund’s complete (initial immunization) or incomplete (subsequent immunizations) adjuvant. Mice received Stx2 toxoid biweekly intervals a minimum of three times.

### *In vitro* Evaluation of Shiga Toxin Production

Vero cells transfected with pStx2 or pGEMT were grown for 48 h. The supernatants were collected and the cells were harvested with 800 µl RPMI and lysed by four freeze-thaw cycles. Serial two-fold dilutions of supernatants or lysates were tested for cytotoxic activity on Vero cells as previously described [48]. Briefly, Vero cells were grown in complete medium on microtiter plates. Aliquots (150 µl) of serial two-fold dilutions of lysates and supernatants were added to each well containing 10^4^ Vero cells. Cells were incubated for 48 h at 37°C in 5% CO_2_. Cells were washed, stained with crystal violet dye, and read on a Microwell System reader 230S (Organon, Teknika, OR) with a 570 nm filter.

The specificity of cytotoxicity was evaluated in parallel, by pre-incubating each sample with mouse anti-Stx2 polyclonal antibody (dilution 1/400) for 1 h at 37°C and for 1 h at 4°C. A positive control with purified Stx2 (1CD50) was carried out.

### Transfection Assays

BHK-21 (Syrian hamster kidney fibroblasts from the American Type Culture Collection) and Vero cells (kidney epithelial cells of the African Green Monkey) were grown in RPMI 1640 medium (EMEVE Microvet SRL Laboratories, Argentina), supplemented with 10% fetal bovine serum (Natocor, Argentina), 100 U/ml penicillin G sodium, 100 µg/ml streptomycin sulphate, and 0.25 µg/ml fungizone (GIBCO, Grand Island, NY, USA) (complete medium) to 4×10^5^ cells/well in six-well culture plates (GBO, Germany), at 37°C in 5% CO_2_. Cells were washed twice with serum-free medium and transfected with Polyfect reagent (QIAGEN Inc, CA, USA). Briefly, 2.5 µg of plasmid pStx2, linear *stx2* or pGEMT (PROMEGA Inc.) religated vector were mixed with 15 µl of Polyfect reagent following the manufacturer’s instructions. After 10 minutes, the cells were incubated with the transfection mix (DNA-polyfect) for 48 h using complete medium at 37°C in 5% CO_2_. The pStx2-transfected cells were analyzed by cytotoxic assay, or fixed for immunofluorescence analysis.

293T cells (human embryonic kidney cells) were transfected with supercoiled or linear peGFP constructions (pr1-eGFP, pr1ΔTATA-eGFP, pr7-eGFP, pr7ΔTATA-eGFP and Δpr-eGFP) as described above. Cells were analyzed by fluorescence microscopy.

### Analysis of Cytoskeletal Actin

BHK cells were grown on cover slips and transfected with pStx2 as previously described. After 48 hours, cells on cover slips were washed with PBS and fixed with PBS containing 1% p-formaldehyde (PFA) for 15 minutes at room temperature. Cells were washed three times with PBS for 5 minutes, permeabilized with PBS containing 0,25% Triton X100 for 15 minutes at room temperature, and washed again three times with PBS for 5 minutes. Cells were stained with phalloidin-TRITC (Sigma-Aldrich, St. Louis, MO, USA) and DAPI (Molecular Probes-Invitrogen, CA, USA) according to the manufacturer’s specifications. The samples were analyzed by fluorescence microscopy in a Nikon Eclipse TE2000 (NIS-Elements imaging software) equipped with a CCD camera.

### Immunofluorescence Assays

Vero cells were grown on cover slips, transfected with pStx2 or pGEMT vector and incubated for 48 h. As positive control, cells were incubated with purified Stx2 (100CD50) for 2 h at 37°C. Cells were washed with PBS, fixed with PBS containing 1% PFA for 15 minutes at room temperature and washed three times with PBS. Cells were permeabilized with 0.25% Triton X100 in PBS for 15 minutes at room temperature, washed again three times with PBS and incubated with block solution (Triton X100 0.25%, BSA 4%, PBS 1X) for 90 minutes at 37°C. Then, cells were incubated with mouse anti-Stx2 polyclonal antibody diluted 1/100 in block solution for 90 minutes at 4°C, washed five times with PBS and incubated with goat anti-mouse IgG−FITC antibody (dilution 1/2000) for 45 minutes at 4°C (Jackson ImmunoResearch Laboratories Inc., PA, USA). Finally, the samples were washed five times with PBS, stained with propidium iodide (PI) (Sigma-Aldrich) (5 µg/ml) and analyzed with FluoViewTM FV1000 Confocal Microscope (Olympus). Excitation wavelengths were 488 nm for FITC and 543 nm for PI and a sequential mode was used. The numerical aperture was 1.42 on a 60X oil objective.

### *In vitro* Evaluation of the Ability of Bacteriophage 933W to Transduce THP-1 Differentiated Cells

C600: ΔTOX-GFP was generously provided by Dr Alison Weiss [14]. It is a non-pathogenic phage resulting from purified 933W bacteriophage in which *stx2* gene was replaced by *gfp* sequence. THP-1 cells were differentiated to macrophages by incubation with phorbol-12-myristate-13-acetate (PMA) during 48 h. Phages at a multiplicity of infection (M.O.I) equal to 1 were added to macrophages. Transduction of macrophages was enhanced by centrifugation at 1000 × g for 10 min at room temperature. After 10 min at 37°C, medium containing phage was removed and cells were washed twice with PBS and then incubated in complete medium. After 3 hours post infection, the cells were examined by FluoViewTM FV1000 Confocal Microscope (Olympus).

### Statistical Analysis

The significance of the difference between treatments was analyzed using (ANOVA with Tukey’s post-test) with Prism 5.0 software (GraphPad Software), and the p value is indicated by asterisks in the figures.

### Ethics Statement

Experiments performed herein were approved by the (IMEX) Instituto de Medicina Experimental Care Committee in accordance with the principles set forth in the Guide for the Care and Use of Laboratory Animals (National Institute of Health, 1985).

## Supporting Information

Figure S1 **GFP activity driven by pr1ΔTATA-eGFP and pr7ΔTATA-eGFP.** 293 T cells were transfected with pr1ΔTATA-eGFP or pr7ΔTATA-eGFP. After 48 h, cells were analyzed by fluorescence microscopy using Nikon Eclipse TE2000 microscope equipped with a CCD camera, using 400X magnification. Green fluorescence photos were taken with 600 ms of exposure and 1 of gain. Numbers 1, 2, 3, 4 correspond to images visualized with white light, green filter, DAPI, merge between white light, green filter and DAPI respectively. **A.** Cells transfected with pr1ΔTATA-eGFP. **B.** Cells transfected with pr7ΔTATA-eGFP.
(TIF)
