# Supplementary figures and images for: Functional Capacity of Shiga-Toxin Promoter Sequences in Eukaryotic Cells
Source: PLoS One. 2013 Feb 22;8(2):e57128. doi: 10.1371/journal.pone.0057128 (PMC3579788; doi:10.1371/journal.pone.0057128)

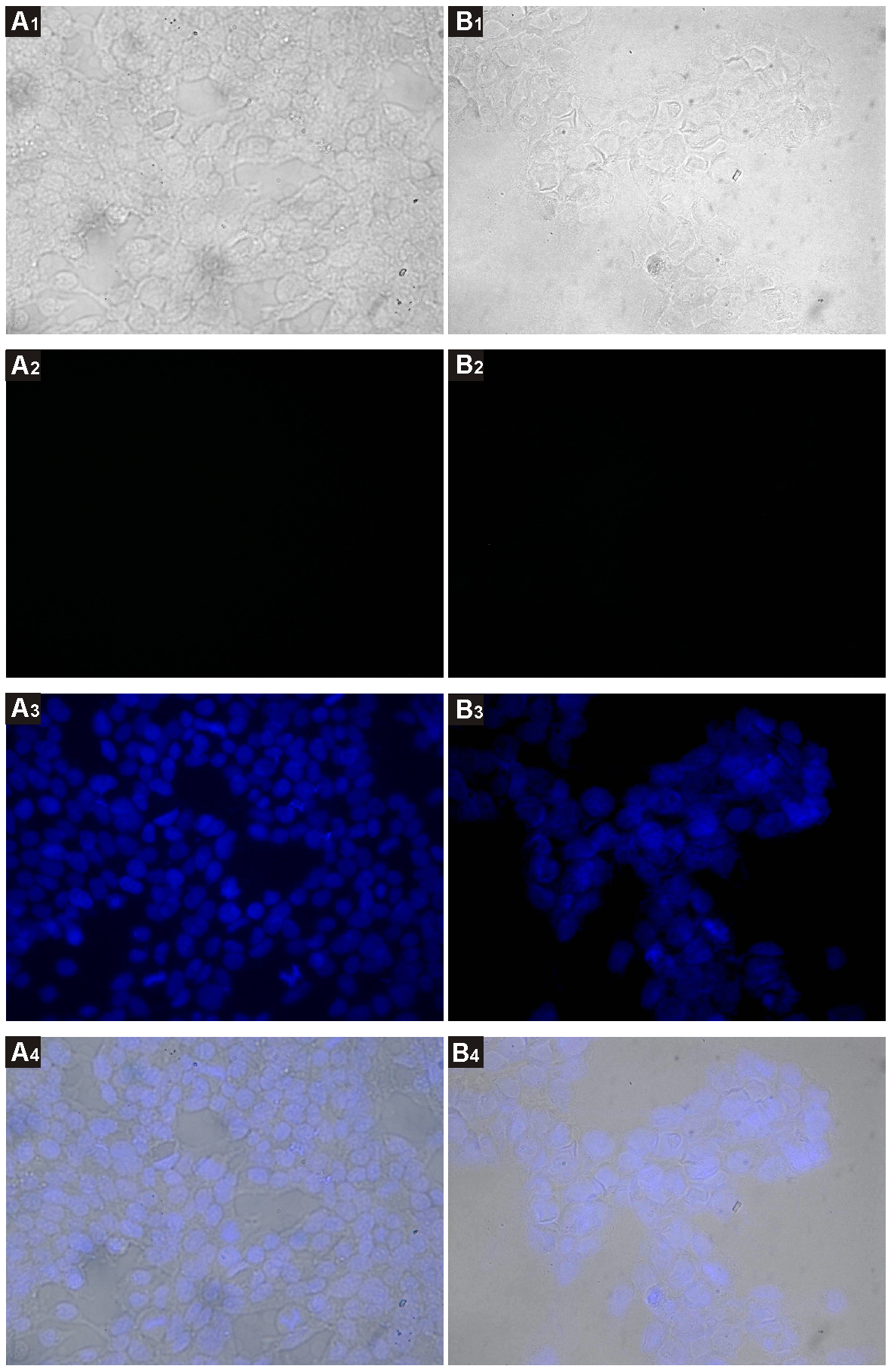

Supplement: Figure S1 — GFP activity driven by pr1ΔTATA-eGFP and pr7ΔTATA-eGFP. 293 T cells were transfected with pr1ΔTATA-eGFP or pr7ΔTATA-eGFP. After 48 h, cells were analyzed by fluorescence microscopy using Nikon Eclipse TE2000 microscope equipped with a CCD camera, using 400X magnification. Green fluorescence photos were taken with 600 ms of exposure and 1 of gain. Numbers 1, 2, 3, 4 correspond to images visualized with white light, green filter, DAPI, merge between white light, green filter and DAPI respectively. A. Cells transfected with pr1ΔTATA-eGFP. B. Cells transfected with pr7ΔTATA-eGFP. (TIF) [file pone.0057128.s001.tif]
